# Evaluation of the relationship between MARCO and CD36 single-nucleotide polymorphisms and susceptibility to pulmonary tuberculosis in a Chinese Han population

**DOI:** 10.1186/s12879-017-2595-2

**Published:** 2017-07-11

**Authors:** Wenting Lao, Hui Kang, Guojiang Jin, Li Chen, Yang Chu, Jiao Sun, Bingqi Sun

**Affiliations:** 1 Department of Laboratory Medicine, The First Affiliated Hospital of China Medical University, Shenyang, Liaoning Province 110001 China; 2 Department of Blood Transfusion, Shanghai East Hospital, Tongji University, Shanghai, 200120 China; 3 Tuberculosis Research Institute of Shenyang Tenth People’s Hospital, Shenyang, Liaoning Province 110041 China

**Keywords:** Pulmonary tuberculosis, Chinese Han population, Single nucleotide polymorphisms, MARCO, CD36

## Abstract

**Background:**

Gene polymorphisms impact greatly on a person’s susceptibility to pulmonary tuberculosis (PTB). Macrophage receptor with collagenous structure (MARCO) and CD36 are two scavenger receptors (SRs) that can recognize *Mycobacterium tuberculosis* (Mtb) and play a key role in tuberculosis infection. Gene polymorphisms of MARCO and CD36 may contribute to tuberculosis risk.

**Methods:**

To investigate whether genetic polymorphisms of MARCO and CD36 are associated with susceptibility to PTB, genomic DNA samples from patients (*n* = 202) and healthy controls (*n* = 216) were collected and analyzed by polymerase chain reaction with high-resolution melting analysis.

**Results:**

We studied two single nucleotide polymorphisms (SNPs) in MARCO (rs12998782 and rs17009726) and three SNPs in CD36 (rs1194182, rs3211956 and rs10499859). Rs12998782 (*P* = 0.018) might be associated with susceptibility to PTB. Rs1194182 (*P* < 0.01) and rs10499859 (*P* < 0.001) might be associated with resistance to PTB. Rs17009726 and rs3211956 were not associated with susceptibility/resistance to PTB.

**Conclusions:**

These data showed that MARCO rs12998782 may increase PTB risk while two SNPs of CD36, rs1194182 and rs10499859 may reduce the risk, indicating MARCO and CD36 as important receptors in response to PTB.

**Electronic supplementary material:**

The online version of this article (doi:10.1186/s12879-017-2595-2) contains supplementary material, which is available to authorized users.

## Background

Tuberculosis (TB) has been a major health problem within human civilizations for thousands of years. After many years struggling against TB, an efficacious regimen has been established that has saved thousands of lives. However, with the appearance of drug resistance as well as an increase in the number of HIV-infected individuals who are susceptible to TB infection, this disease remains a threat. Globally in 2015, an estimated 10.4 million new cases of TB occurred, of which China, India, Nigeria, Indonesia, Pakistan and South Africa accounted for 60% [1]. Though environmental factors and infection status have a major influence on the incidence of TB, evidence indicates that susceptibility or resistance of TB is also determined by genetic factors [2]. Polymorphisms in genes for the Toll-like receptor (TLR), interleukin (IL) [3, 4], vitamin D receptor (VDR) [5] and interferon-gamma (IFN-γ) [6] are associated with susceptibility to tuberculosis.

TB is caused by *Mycobacterium tuberculosis* (Mtb), a pathogenic bacterium that can invade many organs, causing different types of TB, with PTB the most common. Mtb is an intracellular bacterium that mostly infects alveolar macrophages of the host [7]. The cell membrane of macrophages contains SRs that were once thought to be related to the internalization of modified low-density lipoprotein (LDL). However, several studies showed that SRs also recognize several pathogens and play a crucial role in the body’s defense system. MARCO is a class A scavenger receptor that was thought to be upregulated in response to bacterial invasion [8]. Mice deficient in MARCO have shown an impaired ability to eliminate bacteria in the lungs [9]. In human alveolar macrophages, a specific protein that bound inhaled particles and bacteria was established to be MARCO [10]. Later, people used *Mycobacterium marinum*, a close relative of Mtb, to infect zebrafish, and found MARCO knockdown zebrafish suffered an increased bacterial burden [11]. Another study described how mice without MARCO showed a delay in the organization of marginal zone macrophages within the spleen [12]. Furthermore, MARCO recognized trehalose 6,6′-dimycolate (TDM; cord factor), which is one of the most nosogenetic components of Mtb [13].

CD36 is another scavenger receptor and belongs to the class B family of scavenger receptors. CD36 binds many ligands such as oxidized low density lipoprotein, thrombospondin, and certain bacteria, as well as other ligands [14]. Notably, those specific lipids and lipoproteins that make up the main components of mycobacterial organism cell walls are recognized by CD36 [15], [16]. However, CD36^−/−^ mice were found to suffer a reduced mycobacterial burden, indicating CD36 is an important determinant of host susceptibility to mycobacterial infection [17].

MARCO and CD36 are both pattern recognition receptors expressed on macrophages that can recognize Mtb; they connect innate and adaptive immunities. A few studies have investigated the relationship between genetic polymorphisms in MARCO and PTB susceptibility, but similar studies on CD36 are lacking. The aim of our study was to investigate whether gene polymorphisms of MARCO and CD36 are associated with susceptibility to PTB in a Chinese Han population.

## Methods

### Study population

We chose 202 PTB cases from the Tuberculosis Research Institute of Shenyang Tenth People’s Hospital from September 2015 to May 2016. Cases were diagnosed after radiographic examination and were positive for TB cultures or smears. Strain identification was performed after smear positive using gene chip. Patients with cancer, autoimmune disease, HIV and other pulmonary diseases were excluded. The healthy control group consisted of 216 uninfected participants from the Medical Examination Center of The First Affiliated Hospital of China Medical University, which matched cases in the same cohort according to age, sex, race and geographical location. All controls had no history of tuberculosis and were tested normal after blood routine examination, urine routine test, liver function test and radiographic examination. All patients were of Chinese Han descent. The demographic characteristics of study population were listed in Table 1. The gender ratios and mean ages were not significantly different between cases and controls. Details of clinical findings and laboratory examination results about patients and controls were listed in Additional files 1 and 2.

**Table 1:** The demographic characteristics of the study population

	Patients	Controls	*P*
Characteristics			
Sex			0.133
Female	59(29.2%)	78(36.1)	
Male	143(70.8%)	138(63.9)	
Age yrs	47.3 ± 19.4	48.5 ± 13.4	0.44
Smoking			
Smoking	55(27.2%)	ND	
Nonsmoking	127(62.9%)	ND	
Ever Smoking	20(9.9%)	ND	
Laboratory findings			
TP (65-85 g/L)			
< 65	49(24.3%)	21(9.7%)	
65–85	148(73.2%)	195(90.3%)	
ND	5(2.5%)	0	
Alb (40-55 g/L)			
< 40	85(42.1%)	16(7.4%)	
40–55	112(55.4%)	200(92.6%)	
ND	5(2.5%)	0	
TG (0.00–1.70 mm/L)			
0.00–1.70	148(73.3%)	156(72.2%)	
> 1.70	21(10.4%)	60(27.8%)	
ND	33(16.3%)	0	
TC (0.00–5.72 mm/L)			
0.00–5.72	149(73.8%)	187(86.6%)	
> 5.72	20(9.9%)	29(13.4%)	
ND	33(16.3%)	0	
LDL-C (0.00–3.64 mm/L)			
0.00–3.64	146(72.3%)	171(79.2%)	
> 3.64	23(11.4%)	45(20.8%)	
ND	33(16.3%)	0	
HDL-C (0.91–1.92 mm/L)			
< 0.91	78(38.6%)	16(7.4%)	
0.91–1.92	91(45.1%)	189(87.5%)	
> 1.92	0	11(5.1%)	
ND	33(16.3%)	0	
ESR			
Increase	148(73.3%)	ND	
Normal	40(19.8%)	ND	
ND	14(6.9%)	ND	
Clinical phenotype			
Pulmonary tuberculosis	202		
Culture positive	107(53.0%)	ND	
Smear positive	48(23.8%)	ND	
Culture/smear positive	47(23.2%)	ND	
Radiographic findings			
Infiltrative PTB	142(70.3%)	ND	
Cavity PTB	60(29.7%)	ND	
System involvment			
TB pleurisy	22(10.9%)	ND	
Disseminated TB	3(1.5%)	ND	
Extra pulmonary involvement	10(4.9%)	ND	
None	167(82.7%)	ND	
Treatment times			
Initial treatment	98(48.5%)	ND	
Retreatment	104(51.5%)	ND	

ND, Not determined

### DNA isolation

Genomic DNA was extracted from 200 μL of each whole blood sample using QIAamp a DNA Micro Kit (QIAGEN, Hilden, Germany) according to the manufacturer’s instructions. Extracted DNA was diluted to 30 ng/μL and was stored at −80 °C (ThermoFisher Scientific, Bartlesville, OK, USA).

### Single nucleotide polymorphism selection and genotyping

We chose two SNPs of MARCO (rs12998782 and rs17009726) and three SNPs of CD36 (rs1194182, rs3211956 and rs10499859). Rs17009726 was studied in a Chinese Han population by Ma et al. [18]. The SNP, rs12998782, is associated with susceptibility to pulmonary TB in the Gambian population [19], but a similar study has not been conducted in a Chinese Han population. The SNP, rs1194182, was reported to be related to cerebral malaria syndrome [20]. Rs10499859 was included in a study of left ventricular hypertrophy in a Korean population but did not show any significance [21]. As for rs3211956, a study of metabolic syndrome showed it had no association with this SNP [22]. All SNPs were selected from an NCBI dbSNP database (http://www.ncbi.nlm.nih.gov/projects/SNP/) with a minor allele frequency (MAF)> 0.05 in a Chinese Han population.

We used polymerase chain reaction with high-resolution melting analysis (HRM-PCR; Roche Applied Science, Mannheim, Germany) for SNP genotyping. Primer Premier 6 was used to design primers with annealing temperatures ranging from 60 to 65 °C and amplicons were limited to 100–200 bp (Table 2). Real-time PCR cycling and HRM analyses were conducted using a Light Cycler® 480 System (Roche Applied Science). Samples were spiked with wild-type DNA as a standard. Wild-type and mutant homozygotes were distinguished by spiking samples with a known genotype sequence before PCR. Each sample of unknown genomic DNA (30 ng) was used as a template, along with an additional 3.0 ng (rs12998782, rs1194182, rs3211956, rs10499859) of known wild-type DNA. Because only two known genotypes of rs17009726 (wild-type and mutant heterozygous) exist, a supplement of wild-type DNA standard was not needed. PCRs were conducted in 96-well plates using touchdown PCR cycling. HRM curve data were obtained by melting over the range 65–95 °C at a rate of 25 data acquisitions per 1 °C. Results were analyzed using Light Cycler® 480 Gene Scanning software (Roche Applied Science). Forty randomly selected samples were verified by direct sequencing on an ABI7000 sequence detection system (Applied Biosystems, Carlsbad, CA, USA).

**Table 2:** Polymerase chain reaction primers and amplicons

SNP	Direction	Sequence (5′-3′)	Amplicon size (bp)	Annealing temperature (°C)
rs12998782	Forward	AGGAGCTGCAGGTGATAGGAA	118	60.62
	Reverse	GGAGCCCAAGGGAATGTGTG		60.97
rs17009726	Forward	AAACCCACCTGCCCCTATCA	150	60.85
	Reverse	CACTAGCCTGCACTGACCAC		60.67
rs1194182	Forward	CATTTGGCTCAGGTGTCAGG	134	61.65
	Reverse	CACAGGCTCTCAACCCTTCAT		61.59
rs3211956	Forward	CACTTGTGCCAAAGTTGTCC	126	59.19
	Reverse	TACATGCAGCAATCCTGGTC		59.68
rs10499859	Forward	AGTTCTGGGCAAATGTATGTCCT	185	59.99
	Reverse	TGCTTGGCTGGTTAGTTTCCA		60.13

### Statistical analysis

We performed statistics using SPSS 20.0. A Chi-square test was used to analyze genotype and gene allele frequencies in patient and control groups. Odd ratios (ORs) and 95% confidence intervals (CIs) were added to analyze the association between polymorphisms and the risk of tuberculosis. A two-tailed *P* value less than 0.05 was considered statistically significant. All control genotype distributions were in Hardy–Weinberg equilibrium.

## Results

As observed in Table 3, for the rs12998782 polymorphism of the MARCO gene, the presence of a TT homozygous mutation was higher in the patient group than in controls. While for rs17009726, neither allele nor genotype frequencies showed differences between the two groups.

**Table 3:** Distribution of rs12998782, rs17009726, rs1194182, rs3211956 and rs10499859 alleles, and genotype frequencies in PTB patients and healthy controls

	Patients	Controls	OR	χ^2^	95% CI	*P*
	(*n* = 202)	(*n* = 216)				
MARCO rs12998782 Genotype						
CC	117 (57.9%)	131(60.6%)	Reference		Reference	
CT	66 (32.7%)	77 (35.7%)	0.974	0.038	0.750–1.266	0.845
TT	19 (9.4%)	8 (3.7%)	2.427	5.24	1.100–5.357	0.022*
CC + CT	183 (90.6%)	208(96.3%)	Reference		Reference	
TT	19 (9.4%)	8 (3.7%)	2.54	5.617	1.137–5.672	0.018*
Allele						
C	300 (74.3%)	339(78.5%)	Reference		Reference	
T	104 (25.7%)	93 (21.5%)	1.196	2.059	0.936–1.527	0.151
rs17009726 Genotype						
AA	156 (77.2%)	163(75.5%)	Reference		Reference	
AG	46 (22.8%)	53 (24.5%)	0.928	0.18	0.657–1.311	0.671
Allele						
A	358 (88.6%)	379(87.7%)	Reference		Reference	
G	46 (11.4%)	53 (12.3%)	0.928	0.156	0.640–1.345	0.693
CD36 rs1194182 Genotype						
CC	45 (22.3%)	51 (23.6%)	Reference		Reference	
CG	126 (62.4%)	99 (45.8%)	1.116	2.251	0.965–1.291	0.134
GG	31 (15.3%)	66 (30.6%)	0.723	4.497	0.528–0.990	0.034*
CC + CG	171 (84.7%)	150(69.4%)	Reference		Reference	
GG	31 (15.3%)	66 (30.6%)	0.502	13.549	0.343–0.735	<0.01*
Allele						
C	216 (53.5%)	201(46.5%)	Reference		Reference	
G	188 (46.5%)	231(54.5%)	0.87	4.019	0.759–0.998	0.045*
rs3211956 Genotype						
GG	8 (4.0%)	9(4.1%)	Reference		Reference	
GT	58 (28.7%)	56 (25.9%)	1.02	0.086	0.894–1.164	0.769
TT	136(67.3%)	151(70.0%)	1.001	0.001	0.947–1.057	0.979
Allele						
G	74 (18.3%)	74 (17.1%)	Reference		Reference	
T	330 (81.7%)	358(82.9%)	0.986	0.202	0.925–1.050	0.653
rs10499859 Genotype						
AA	79 (39.1%)	57 (26.4%)	Reference		Reference	
AG	109 (54.0%)	102(47.2%)	0.904	1.377	0.764–1.069	0.241
GG	14 (6.9%)	57 (26.4%)	0.301	27.756	0.180–0.505	<0.001*
AA + AG	188 (93.1%)	159(73.6%)	Reference		Reference	
GG	14 (6.9%)	57 (26.4%)	0.263	28.028	0.151–0.456	<0.001*
Allele						
A	267 (66.1%)	216(50.0%)	Reference		Reference	
G	137 (33.9%)	216(50.0%)	0.678	22.152	0.575–0.800	<0.001*

*OR* odds ratio, *95% CI* 95% confidence interval

*P< 0.05 indicates statistical significance

As for rs1194182, the frequency of the G allele was slightly higher in the control group than in patients, while the GG homozygous mutation was significantly higher in the control group (CC + CG vs. GG). Furthermore, both the G allele and the GG homozygous mutation of rs10499859 showed a significant increase in the control group (*P*< 0.001) than in patients. However, a difference was not observed in rs3211956 between the two groups.

Taken together, our results suggest that polymorphisms of MARCO at rs12998782 might be associated with susceptibility to PTB, while polymorphisms of CD36 at rs1194182 and rs10499859, respectively, might be associated with protection against PTB.

We then combined 12,998,782 (from MARCO) with rs1194182 (from CD36), and rs12998782 (from MARCO) with 10,499,859 (from CD36; Table 4), to obtain nine combination genotypes, respectively. Interestingly, we found when two homozygous mutations appeared together (TT of rs12998782 and GG of rs1194182, or TT of rs12998782 and GG of rs10499859), a significant difference was not observed between patients and controls, thus indicating the opposite function of these polymorphisms in MARCO and CD36.

**Table 4:** Distribution of combinations of rs12998782 and rs1194182, or rs12998782 and rs10499859 genotype frequencies in PTB patients and healthy controls

		Patients	Controls				
		(*n* = 202)	(*n* = 216)	OR	χ2	95% CI	*P*
rs12998782	rs1194182						
CC	CC	29 (14.3%)	31 (14.4%)	Reference		Reference	
CT	CC	14 (6.9%)	16 (7.4%)	0.956	0.022	0.532–1.718	0.881
TT	CC	2 (1.0%)	4 (1.9%)	0.565	0.075	0.111–2.873	0.785
CC	CG	70(34.7%)	60(27.8%)	1.072	0.5	0.883–1.303	0.48
CT	CG	43(21.3%)	37(17.1%)	1.098	0.403	0.882–1.456	0.526
TT	CG	13 (6.4%)	2 (0.9%)	5.107	7.156	1.238–21.069	0.007*
CC	GG	18 (8.9%)	40(18.5%)	0.68	3.682	0.448–1.031	0.055
CT	GG	9 (4.5%)	24(11.1%)	0.543	3.908	0.285–1.034	0.048*
TT	GG	4 (2.0%)	2 (0.9%)	2	0.183	0.393–10.181	0.669
rs12998782	rs10499859						
CC	AA	43(21.3%)	39(18.1%)	Reference		Reference	
CT	AA	31(15.3%)	17 (7.9%)	1.38	1.821	0.855–2.228	0.177
TT	AA	5 (2.5%)	1 (0.5%)	4.167	1.087	0.507–34.218	0.297
CC	AG	66(32.7%)	57(26.4%)	1.02	0.029	0.815–1.276	0.864
CT	AG	32(15.8%)	40(18.5%)	0.843	0.981	0.599–1.185	0.322
TT	AG	11 (5.4%)	5 (2.3%)	1.793	1.44	0.673–4.773	0.23
CC	GG	8 (4.0%)	35(16.2%)	0.332	13.369	0.168–0.655	<0.001*
CT	GG	3 (1.5%)	20 (9.2%)	0.192	11.324	0.061–0.608	0.001*
TT	GG	3 (1.5%)	2 (0.9%)	1.337	0.108	0.235–7.609	0.742

*OR* odds ratio, *95% CI* 95% confidence interval

*P< 0.05 indicates statistical significance

**Fig. 1:**
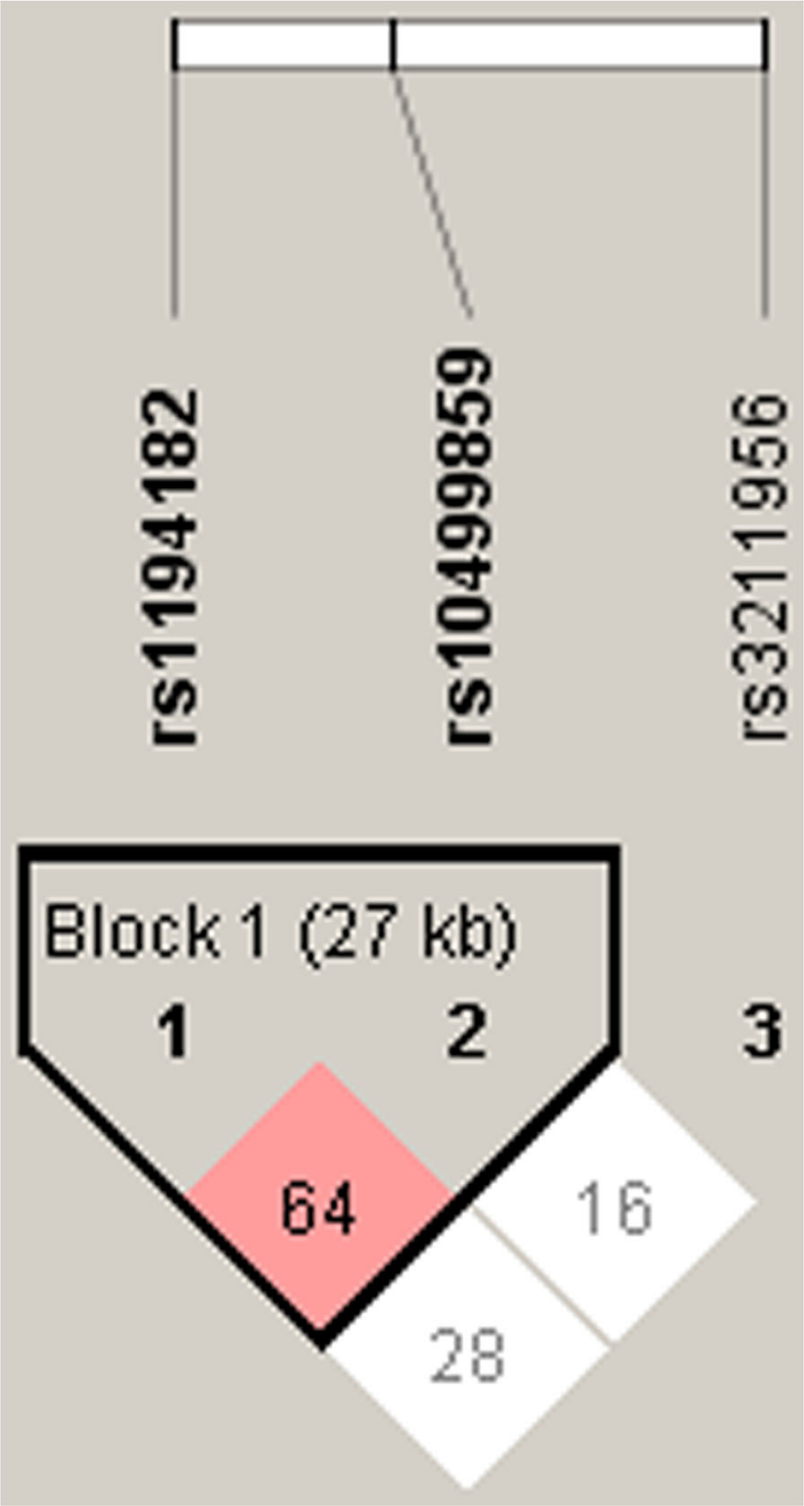
Pairwise linkage disequilibrium (LD) pattern of CD36 region

**Fig. 2:**
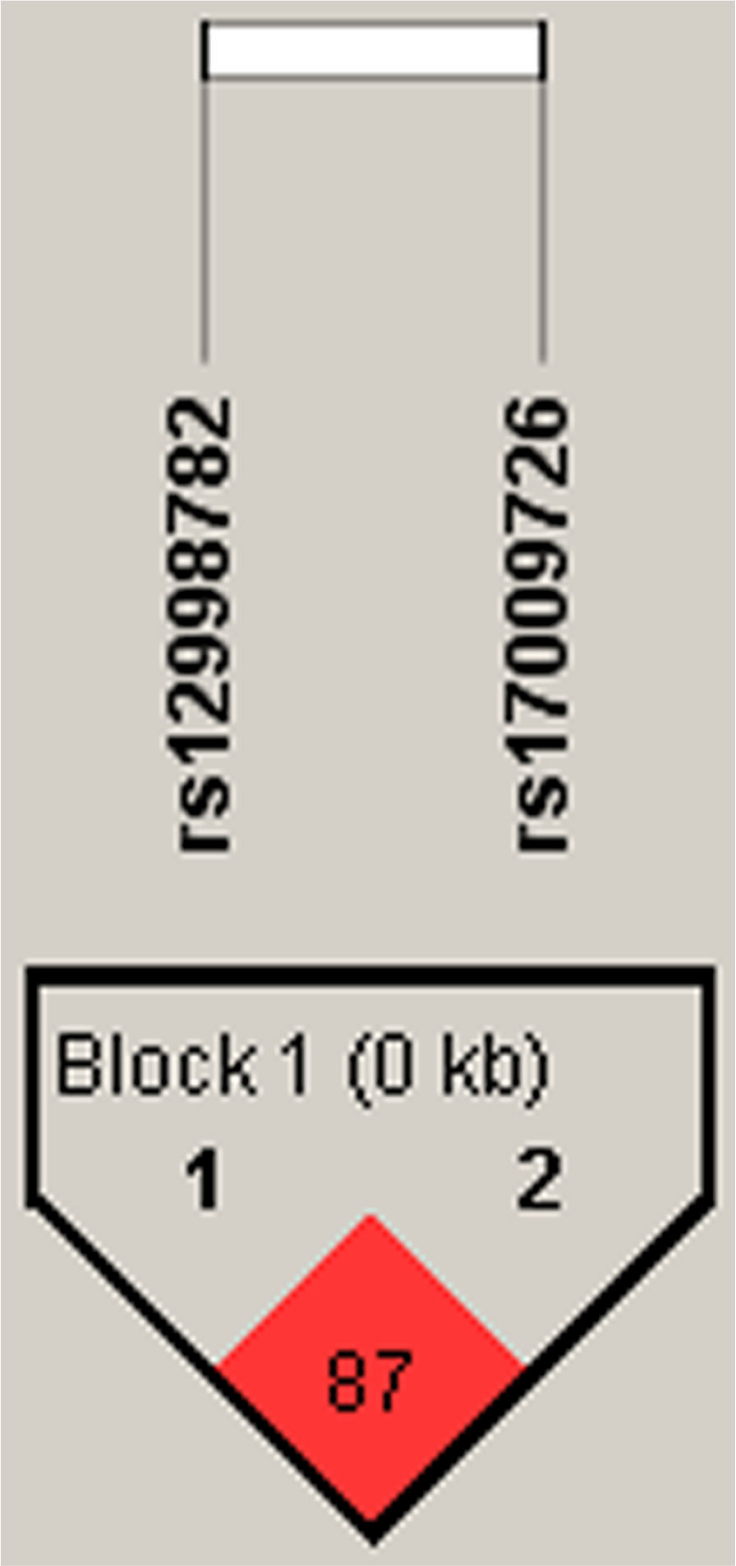
Pairwise linkage disequilibrium (LD) pattern of MARCO region

Then we estimated the LD block using a Haploview version 4.2. Pairwise LD was calculated by both *D’* and r^2^ for the two MARCO SNPs and three CD36 SNPs. The two CD36 variants (rs1194182 and rs10499859) were in strong LD (*D’* = 0.646; r^2^ = 0.302) (Fig. 1). Also the two MARCO variants (rs12998782 and rs17009726) were in strong LD (*D’* = 0.877; r^2^ = 0.335) (Fig. 2). Further, we examined whether haplotypes of CD36 and MARCO were associated with PTB. For CD36, frequencies of haplotype CA were significantly higher in PTB group as compared to the control group and it may confer a higher risk of susceptibility effect for PTB. While the haplotype GG and CG played protective effect for PTB, the frequencies of haplotype GG and CG were significantly higher in control group (Table 5). However, none of the haplotypes of block1 in MARCO showed any significant difference between PTB and control group (datas not shown).

**Table 5:** Haplotype frequencies constructed with SNPs in the PTB group and the control group (rs1194182, rs10499859)

Haplotype	Frequency	Case (*n* = 202)	Controls (*n* = 216)	χ^2^	*P*
CA	0.424	0.504(203.6)	0.35(151.2)	20.261	<0.001*
GG	0.347	0.308(124.6)	0.382(165.2)	5.044	0.0247*
GA	0.155	0.157(63.4)	0.152(65.8)	0.034	0.8541
CG	0.074	0.031(12.4)	0.115(49.8)	21.699	<0.001*

*P< 0.05 indicates statistical significance

As nutrition status can affect the susceptibility to PTB, we performed stratified analysis according to albumin (ALB) levels (Table 6). We found in normal-ALB group (ALB> 40g/L), still TT of rs12998782 was significantly higher in PTB group than control group. Also GG of rs1194182 and rs10499859 showed an significant increase in control group. While in the low-ALB group, there was no difference in distribution of any genotypes or alleles between PTB group and control group.

**Table 6:** Stratified analysis of distribution of rs12998782, rs1194182 and rs10499859 alleles, and genotype frequencies in PTB patients and healthy controls according to the levels of albumin

	ALB < 40(g/L)						ALB > 40(g/L)					
	Patients	Controls	OR	χ^2^	95% CI	*P*	Patients	Controls	OR	χ^2^	95% CI	*P*
	(*n* = 202)	(*n* = 216)					(*n* = 202)	(*n* = 216)				
MARCO rs12998782 Genotype												
CC	50	11					64	120				
CT	28	5	1.232	0.126	0.389–3.906	0.723	36	72	0.952	0.036	0.576–1.575	0.849
TT	7	0	−	−	−	−	12	8	2.813	4.907	1.094–7.233	0.027*
CC + CT	78	16					100	192				
TT	7	0	−	−	−	−	12	8	2.88	5.395	1.140–7.275	0.02*
Allele												
C	78	16					100	192				
T	92	16	1.179	0.194	0.554–2.511	0.688	124	208	1.145	0.65	0.824–1.59	0.42
CD36 rs1194182 Genotype												
CC	18	3					26	49				
CG	53	8	1.104	<0.0001	0.246–4.617	1	70	92	1.434	1.553	0.812–2.531	0.213
GG	15	5	0.5	0.222	0.102–2.444	0.638	16	61	0.494	3.664	0.239–1.023	0.056
CC + CG	71	11					96	141				
GG	15	5	0.465	0.873	0.141–1.535	0.35	16	61	0.385	9.857	0.21–0.708	0.002*
Allele												
C	89	14					122	190				
G	83	18	0.725	0.69	0.339–1.551	0.406	102	214	0.742	3.186	0.535–1.03	0.074
rs10499859 Genotype												
AA	35	5					42	52				
AG	42	7	0.857	0.06	0.25–2.939	0.806	64	95	0.834	0.476	0.498–1.397	0.49
GG	8	4	0.286	2.799	0.062–1.31	0.094	6	53	0.14	20.052	0.055–0.385	<0.001*
AA + AG	77	12					106	147				
GG	8	4	0.312	3.125	0.081–1.197	0.077	6	53	0.157	20.929	0.065–0.379	<0.001*
Allele												
A	112	17					148	199				
G	58	15	0.587	1.899	0.274–1.259	0.168	76	201	0.508	15.495	0.362–0.714	<0.001*

*OR* odds ratio, *95% CI* 95% confidence interval

*P< 0.05 indicates statistical significance

## Discussion

Our results indicate that polymorphisms in the human class A scavenger receptor, MARCO, and the class B scavenger receptor, CD36, are associated with susceptibility/resistance to PTB in a Chinese Han population. According to our study, the TT genotype of rs12998782 in the *MARCO* gene can increase the risk of tuberculosis. This is consistent with findings from a recent study of a Gambian population that revealed rs12998782 was associated with susceptibility to PTB [19]. However, genotypes or gene alleles of rs17009726 showed no significant difference between cases and controls, which did not coincide with the results of a previous study on a Chinese Han population performed by Ma et al. [18]. Then we discussed separately according to age and sex, there was still no significant difference between PTB group and control group. Also we discussed according to treatment times (Initial treatment of PTB and retreatment of PTB), no difference was observed.

The *MARCO* gene is located on 2q14.2, and rs12998782 is located within intron 1. Though intron variants are not responsible for changes in amino acids, they may influence the transcription and splicing process. Studies on certain genes have revealed that intron 1 is essential for the full expression of rat genes [23, 24]. Thus, a variant in rs12998782 may impact the expression of *MARCO* on human alveolar macrophages, which may fail to recognize Mtb efficiently and may be defective in cooperating with TLR2/TLR4 [13]. A recent study found that it is the reduced phagocytotic ability of pathogens, rather than reduced cytokine production, that explains the association between *MARCO* polymorphisms and susceptibility to PTB [25]. A decreased ability to phagocytose Mtb will lead to a reduction of its clearance, which would increase susceptibility to PTB. However, due to a disparity between the experimental model (mice) and our investigated patients (human), mice with a *MARCO* knockout may represent a more notable defect in responding to Mtb [13], while for humans, *MARCO* polymorphisms may only have a slight impact.

On the other hand, we found the GG genotypes of rs1194182 and rs10499859 in the CD36 gene are associated with resistance to PTB. The CD36 gene is located on 7q21.11; rs1194182 is located in the 5′ untranslated region (5′ UTR), while rs10499859 is in intron 1. The 5′ UTR is a major site of translational regulation, a key step in eukaryotic gene expression [26]. Single nucleotide polymorphisms in the 5′ UTR of the CD36 gene may affect its expression and the function of the protein. Thus, the GG homozygous mutation of rs1194182 may lead to the dysfunction of CD36. Interestingly, this result is consistent with a study performed by Hawkes et al., which demonstrated that CD36^−/−^ mice suffered fewer mycobacterial infections than CD36^+/+^ mice [17]. CD36 participates in the process of apoptosis and Mtb infected macrophages undergoing apoptosis can be phagocytosed by new macrophages induced by intracellular Mtb, thus leading to the expansion of infection [27, 28]. In other words, CD36 may cooperate with Mtb in infection. Hence, this may explain the homozygous mutations of rs1194182 and rs10499859 acting as protective factors against PTB.

We used Mutation Taster (http://www.mutationtaster.org/) to predict possible function changes of rs12998782 and rs10499859. We found these two SNPs were low conservative (PhyloP = −0.559, PhastCons = 0.008; PhyloP = −0.656, PhastCons = 0) and would lead to splice site changes and protein features might be affected.

As for the combination of MARCO and CD36, no significant difference was observed between patients and controls when two homozygous mutations from MARCO and CD36, respectively, coexisted. This indicates that polymorphisms of these two genes may have opposite effects on the susceptibility to PTB.

To date, this is the first time that the relationship between CD36 SNPs (rs1194182 and rs10499859) and PTB has been analyzed. For MARCO, Ma et al. [18] identified several SNPs that correlated with TB in a Chinese Han population; however, rs12998782 was not included in these studies.

One limitation of our study was the insufficient information available concerning patients and controls: More stratified analyses are required to make our study more enriched. In addition, extra-pulmonary tuberculosis also deserves attention. A recent study revealed two SNPs of MARCO, rs2278589 and rs6751745, were associated with PTB but not with TB meningitis (TBM) [25]. Whether MARCO and CD36 are associated with extra-pulmonary tuberculosis, such as TBM, or intestinal, bone and urinary tuberculosis, needs further investigation.

## Conclusions

In summary, our findings demonstrated that genetic variations in the scavenger receptors, MARCO and CD36, might be associated with susceptibility/resistance to pulmonary tuberculosis in a Chinese Han population. It remains to be determined whether genetic variations in MARCO and CD36 alter the expression of these receptors and whether genetic polymorphisms impact the severity of infection and the persistence of Mtb over a longer time period.

## Additional files

Additional file 1: Table S1. PTB patients information. Some laboratory examination results and clinical findings were listed. (XLSX 32 kb)

Additional file 2: Table S2. Controls information. Some laboratory examination results and clinical findings were listed. (XLSX 33 kb)

## Abbreviations

CD36: Cluster of differentiation 36
HRM-PCR: Polymerase chain reaction with high-resolution melting analysis.
MAF: Minor allele frequency
MARCO: Macrophage receptor with collagenous structure
Mtb: *Mycobacterium tuberculosis*
PTB: Pulmonary tuberculosis
SNP: Single nucleotide polymorphism
